# Linking classification and therapeutic management of vasculitides

**DOI:** 10.1186/s13075-015-0654-x

**Published:** 2015-06-02

**Authors:** Corisande Baldwin, Simon Carette, Christian Pagnoux

**Affiliations:** Division of Rheumatology, Department of Medicine, University of British Columbia, 1200 Burrard Street, Vancouver, BC V6Z 2C7 Canada; Division of Rheumatology, Department of Medicine, Mount Sinai Hospital, 60 Murray Street, Toronto, Ontario M5T 3L9 Canada

## Abstract

Vasculitides are classified by the size, type and location of the predominantly involved vessels and by their primary or secondary nature. Their treatment depends on the type of vasculitis, its etiology (when known), and its severity and must be further adjusted by the individual characteristics and comorbidities of patients. In this paper, we review how the classification and definition of vasculitides have evolved over the past years and how it has affected therapeutic changes. As new genetic markers are being discovered and the pathogenesis of vasculitides continues to be elucidated, further modifications in classification and treatment can be expected.

## Introduction

Vasculitides are heterogeneous diseases characterized by the inflammation of blood vessels. Clinical manifestations differ by the size, type and location of vessels involved. Disease definitions and classifications have evolved over time with a better understanding of pathogenesis [1–4]. Further modifications are expected as pathogenic mechanisms are elucidated [5–8]. Here, we review how the current classifications and definitions of vasculitides differ from earlier versions and how these changes have affected treatment.

## Current classifications, nomenclature and definitions of vasculitides

With the exception of Takayasu arteritis (TAK), Kawasaki disease, polyarteritis nodosa (PAN) and Behçet disease [9–11], we lack consensual and widely validated diagnostic criteria for most of the vasculitides.

### 1990 American College of Rheumatology classification

In 1990, the American College of Rheumatology (ACR) published criteria for the classification of vasculitides. Separate criteria were developed for giant cell arteritis (GCA), TAK, PAN, Wegener’s granulomatosis (now granulomatosis with polyangiitis (GPA)), Churg-Strauss syndrome or allergic granulomatosis and angiitis (now eosinophilic granulomatosis with polyangiitis (EGPA)), hypersensitivity vasculitis and Henoch-Schonlein purpura (now immunoglobulin A (IgA) vasculitis). These criteria were based on the analysis of patients with definite vasculitis and assigned a specific vasculitis diagnosis according to their physician’s opinion. The aim was to standardize the evaluation and description of patients with vasculitis. This classification has been useful in distinguishing between forms of vasculitis and is still often used for research. The sensitivity and specificity of the ACR criteria range from 71.0 to 95.3 % and 78.7 to 99.7 %, respectively, depending on the disease. Many of the other types of vasculitides lacked criteria; one example is microscopic polyangiitis (MPA), considered at the time to be a ‘microscopic’ form of PAN. In addition, the ACR criteria did not incorporate testing for antineutrophil cytoplasm antibodies (ANCAs), discovered in the mid-1980s. They also did not take into account the underlying disease pathophysiology, largely unknown at the time the criteria were developed.

### International Chapel Hill Consensus Conference nomenclature of vasculitides

In 1994, the Chapel Hill group held an international conference to develop a nomenclature for vasculitides [1]. These disease definitions were not intended as classification criteria and even less so as diagnostic criteria. The 1994 Chapel Hill Consensus Conference (CHCC) nomenclature distinguished vasculitides by the size of the vessels predominantly affected. Large-sized-vessel vasculitides included GCA and TAK; medium-sized-vessel vasculitides included PAN and Kawasaki disease; small-sized-vessel vasculitides were subtyped into Wegener’s granulomatosis (now GPA), MPA, Churg-Strauss syndrome (now EGPA), Henoch-Schonlein purpura (now IgA vasculitis), essential cryoglobulinemic vasculitis, and cutaneous leukocytoclastic vasculitis. Compared with the ACR criteria, the proposed CHCC nomenclature did not incorporate ‘hypersensitivity vasculitis’ but added definitions for Kawasaki disease and essential cryoglobulinemic vasculitis. They also defined MPA as a new entity, separate from PAN. The 1994 CHCC nomenclature mentioned, but only as footnote, the frequent association of ANCAs and Wegener’s granulomatosis, MPA and Churg-Strauss syndrome.

The CHCC nomenclature and definitions were revised in 2012 to incorporate updated knowledge of the etiology, demographics, pathogenesis, pathology and clinical manifestations of vasculitides [2]. In contrast to the previous version, the revision divided vasculitides by vessel size and etiology (primary vasculitides versus vasculitides associated with other systemic diseases or probable etiologies, including infectious or drug-induced vasculitis). It also added definitions for vasculitides affecting vessels of variable sizes, such as Behçet disease or Cogan’s syndrome, as well as single-organ vasculitides, such as central nervous system (CNS) vasculitis. This revised nomenclature phased out most of the eponyms by replacing Wegener’s granulomatosis with GPA, Churg-Strauss syndrome with EGPA and Henoch-Schonlein purpura with IgA vasculitis. Vasculitides of small-sized vessels were further subdivided into ANCA-associated vasculitides (encompassing GPA, MPA, and EGPA; thereby including ANCA test results and specificity as an important disease characteristic) and immune-complex small-vessel vasculitides (encompassing anti-glomerular basement membrane disease (anti-GBM disease), cryoglobulinemic vasculitis, hypocomplementemic urticarial vasculitis/anti-C1q vasculitis, and IgA vasculitis).

### 2007 European medicines evaluation agency classification algorithm

In a previous attempt to address the limitations of the 1990 ACR classification criteria, the Medicines Evaluation Agency (EMA) developed an algorithm for classifying ANCA-associated vasculitides and PAN in epidemiological studies [4]. Rather than devising new criteria, the aim of the EMA classification algorithm was to achieve consensus on the application of both the 1994 CHCC definitions and the ACR criteria. In this algorithm, MPA was distinguished as a separate disease entity and ANCA test results played an important role. Despite its practicability and validation in separate cohorts and even using the 2012 instead of 1994 CHCC definitions [12], the EMA classification algorithm is used infrequently and does not address the other vasculitic entities.

### Pediatric criteria for vasculitis

Because the 1990 ACR criteria were based on adult rather than pediatric populations, in 2010 the European League Against Rheumatism (EULAR), the Paediatric Rheumatology International Trials Organization and the Paediatric Rheumatology European Society proposed a different set of classification criteria for pediatric patients with Henoch-Schonlein purpura (now IgA vasculitis), childhood-PAN, childhood-Wegener’s granulomatosis and childhood-TAK [13]. These classification criteria did not include Kawasaki disease, the most common vasculitis in children, nor rarer vasculitides such as cryoglobulinemic vasculitis or EGPA. Kawasaki disease is the subject of separate ongoing evaluation studies. Importantly, in this article, we focus more on adult populations; readers can refer to multiple other articles on pediatric vasculitis [14–17].

## Evidence for different disease groupings or additional disease subtypes and therapeutic consequences

The treatment of vasculitides depends on the specific type of vasculitis according to the CHCC nomenclature, its etiology when known (primary versus secondary, such as due to drugs or infections) and severity. Therapeutic decisions should also take into consideration current knowledge of disease outcomes such as the risk of relapse and the possible short- and long-term complications, the disease itself or its treatments, as well as individual patient characteristics and comorbidities.

Because the revised 2012 CHCC nomenclature has been widely accepted as an international consensus, its authors implicitly acknowledged that changes may become justified in the future, although they also warned against any precipitation in doing so (‘As in the original CHCC, the emphasis was on making changes only when justified’). Since its publication, several alterations and the addition of other vasculitis subsets that do not really fit with any of the CHCC-defined conditions have already been suggested [7, 18, 19].

In the next sections, we review the main vasculitis subtypes described in the CHCC nomenclature, review the evidence supporting the distinction and/or addition of separate disease subtypes, and discuss possible therapeutic implications.

### Primary large-sized-vessel vasculitides

TAK and GCA may represent two clinical variants of the same disease, with differences related to patient age, geographical area, genetic background and/or other yet unidentified factors. In both diseases, histology of tissue demonstrates chronic granulomatous inflammation. GCA is the most commonly diagnosed vasculitis in adults older than 50 years, whereas TAK is less common and affects mostly women younger than 40 years. GCA is the most common in regions of predominantly Scandinavian ancestry, and TAK is more often observed in Asian populations. Genetic associations differ: GCA is associated with HLA-DRB1*04 and TAK with HLA-B*52:01 and HLA-B*67:01 polymorphisms, at least in Asian and Turkish patients [20, 21]. Clinically, GCA predominantly affects the branches of the external carotid artery, resulting in classic temporal arteritis, whereas TAK predominantly involves the aorta and its major branches, including the subclavian and carotid arteries [22, 23].

Treatments for both GCA and TAK ultimately rely on glucocorticoids and, when relapsing or refractory, the addition of another immunosuppressive drug such as methotrexate [22, 23]. We have more data on the use of methotrexate for GCA than TAK [22, 23]. Methotrexate has been shown to moderately reduce the risk of GCA relapse as well as the cumulative glucocorticoid dose and to induce remission in patients with TAK refractory to corticosteroids. Leflunomide has shown partial efficacy in a case series of GCA patients. Cyclophosphamide can be used as a third-line agent for severe disease, with failure of the aforementioned drugs. A few case reports suggest some efficacy for azathioprine and mycophenolate mofetil in patients with GCA and for mizoribine and tacrolimus in TAK [22, 23].

Early case reports suggested the benefit of anti-tumor necrosis factor alpha (anti-TNFα) agents for GCA, but three subsequent randomized controlled trials (of infliximab, adalimumab and etanercept) failed to confirm this benefit [24–26]. Anti-TNFα agents are thought to be more effective for TAK, although this conclusion results only from retrospective series [27]. Case series have suggested the benefit of interleukin-6 blockade with tocilizumab for both GCA and TAK [28]. Tocilizumab is currently being investigated for GCA in randomized controlled trials (the international GiACTA study (ClinicalTrials.gov identifier: NCT01791153) and a smaller monocentric study (NCT01450137)) [29]. Aside from case reports and a small series of TAK patients, we have minimal evidence for the benefit of rituximab for GCA or TAK [27].

Identifying GCA or TAK subsets that would benefit from different treatment approaches is probably not the most urgent issue to tackle compared with finding more effective and safer treatments. Although TAK can be further categorized by the anatomical distribution of arterial lesions, such a categorization has no obvious association with its medical treatment [30]. The choice of vascular treatment for TAK lesions (surgery versus endovascular) varies by the artery needing treatment. Angioplasty and stenting are acceptable for focal stenosis of renal, aorta and iliac arteries, but for most other lesions, the risk of complications, including aneurysms and re-stenosis, seems lower with surgery [31, 32].

Other inflammatory large-sized-vessel entities, some of them considered variants of TAK or GCA, have been described over the past decades. Patients with typical GCA and temporal arteritis may have extra-cranial large-vessel involvement, including the aorta (3 to 18 % at diagnosis, mainly the ascending and thoracic aorta, and up to 50 % if based on fluorodeoxyglucose-positron emission tomography scan investigations) and limb arteries (up to 30 % of patients based on Doppler-ultrasonography studies) [33]. Large-sized vessel involvement can also occur without the typical cranial involvement in patients older than 50 years, and eventually closely resembles TAK (sometimes called large-vessel GCA (LV-GCA)). A recent retrospective study demonstrated reduced time to relapse, increased rate of relapses, increased overall glucocorticoid exposure, and increased rate of aortic aneurysm development in patients with LV-GCA compared with GCA patients without extra-cranial large vessel disease [34, 35]. The involvement of the aorta can lead to aortic aneurysm, typically occurring more than 5 years after the diagnosis, often when the patient is off medications, and is associated with increased mortality (standardized mortality ratio 2.63 (95 % confidence interval, 1.78 to 3.73)) [34]. The treatment approach for LV-GCA remains similar to that for GCA limited to cranial arteries.

Isolated aortitis and IgG4-related aortitis are other large-sized-vessel inflammatory conditions. Isolated aortitis may simply be part of the spectrum of GCA or TAK but in a more localized form, and long-term studies are needed to better answer this question. Patients with isolated aortitis are commonly asymptomatic or have non-specific symptoms, so the true prevalence of the disease is difficult to estimate. In contrast to GCA, isolated aortitis is seen more often in men, in their sixth decade [36].

IgG4-related aortitis most often presents as an inflammatory aneurysm, chiefly of the abdominal and less commonly the thoracic aorta, although it can sometimes be present in other arteries [37, 38]. The aortic wall is usually not as thickened as in isolated aortitis or aortitis due to other etiologies; it can dissect and the inflammation seems to predominate in the adventitia rather than the intima-media. The diagnosis relies on the combination of clinical findings, elevated serum levels of IgG4, and, most reliably, histology [39]. The response to glucocorticoids in IgG4 aortitis is usually good, but for refractory or relapsing cases, rituximab has yielded some promising results [40].

### Medium-sized-vessel vasculitides

Kawasaki disease is a disease of childhood affecting boys more than girls with a mean age at diagnosis of 2 years. The disease is manifested as fever and lethargy associated with conjunctival injection (80 to 90 %), muco-cutaneous changes (cheilitis, pharyngitis, ‘strawberry tongue’ in 80 to 90 %), erythema and edema of the hands and feet with secondary periungal desquamation (80 %), polymorphous, non-vesicular truncal rash (>90 %), and cervical lymphadenopathy (50 %). Incomplete forms of Kawasaki disease have been described in adults and rare complete forms in subjects infected with HIV [41]. While the disease is self-limited in time, coronary artery aneurisms may complicate the disease process and are associated with mortality rates of over 1 % if untreated [42]. As for children, the cornerstone treatment in adults remains prompt initiation of aspirin and intravenous Ig (IVIg) at a dose of 2 mg/kg. IVIg significantly reduces the risk of coronary artery aneurysms [43]. Patients who remain or re-develop fever within the first 48 hours after IVIg should receive repeated dosing. The role of glucocorticoids is controversial and should be considered in IVIg-resistant patients (10 to 15 % of the patients) and as first-line therapy combined with IVIg in those presenting myocarditis or shock. Increasing evidence suggests that they may be helpful in patients with other severe forms, although the definition of these severe forms, as identified in Japanese patients, may not apply to other populations [41, 44]. Retrospective series suggested the efficacy and safety of TNF-α inhibitors for Kawasaki disease, but a recent controlled trial with infliximab failed to confirm any such major efficacy [45–47].

PAN is a necrotizing arteritis involving predominantly medium-sized vessels. In the 1994 CHCC nomenclature, hepatitis B virus (HBV)-related PAN was not clearly individualized. In the revised 2012 CHCC nomenclature, HBV-related PAN is categorized with vasculitides associated with probable etiologies [1, 2]. Intriguingly, the incidences of both HBV- and non-HBV-related PAN have decreased markedly since the availability of vaccine against HBV in the mid-1990s [48, 49]. The initial treatment of non-HBV-related PAN relies on glucocorticoids, combined with an immunosuppressive agent, mainly cyclophosphamide, in most severe forms, followed by maintenance (for example, with azathioprine or methotrexate), although relapses are not that common [48]. The treatment of HBV-related PAN relies on the combination of antiviral agents to eradicate the virus (currently mostly entecavir or tenofovir), plasma exchange to remove circulating immune complexes, and a much shorter course of glucocorticoids (about 2 weeks) to rapidly control the vasculitic manifestations without increasing viral replication [48, 50].

The etiology(ies) of a few other PAN-like conditions elucidated recently include recessive loss-of-function mutations in adenosine deaminase 2 causing a vasculopathy with necrotic cutaneous manifestations, early-onset stroke, renal and/or gastrointestinal ischemia and infarct. This vasculopathy tends to respond better to anti-TNFα, rather than to other immunosuppressive agents, including cyclophosphamide [51, 52]. Other mutations will likely be identified in the future.

### Small-sized-vessel vasculitis

Most changes in the classification and treatment of vasculitides in the past decades have related to small-sized vessel vasculitides. Additional changes have already been suggested [7, 53, 54].

The 2012 CHCC nomenclature clearly separated ANCA-associated (GPA, MPA and EGPA) from immune-complex small-sized-vessel vasculitides [2]. Both subsets demonstrate necrotizing vasculitis of small-sized vessels on histology, but ANCA-associated vasculitides are classically ‘pauci-immune’, with a relative absence of immune deposits, at least when compared with the other immune-complex vasculitides. Immune deposits have indeed been reported in pulmonary and skin lesions of GPA patients, as well as on renal histology [55–57]. More common and prominent Ig depositions can be observed in vessel walls in immune-complex small-sized-vessel vasculitides. Both small-sized-vessel vasculitis subsets also differ in their pathogenesis, clinical manifestations and therapeutic strategies (although most of the drugs used to treat them are the same).

GPA, MPA and EGPA share some similarities, including ANCAs in some patients. However, the strength of this association varies among the three conditions: 80 to 90 % of cases of systemic GPA or MPA are ANCA-positive compared with only 30 to 40 % of cases of EGPA. ANCA type and specificity may vary by geography. In North American and European populations, ANCAs in patients with GPA are mostly the c-ANCA type (with a cytoplasmic labeling pattern on immunofluorescence) and show proteinase 3 (PR3) specificity on enzyme-linked immunosorbent assay (ELISA), whereas patients with MPA and EGPA show predominantly p-ANCA (with a perinuclear labeling pattern on immunofluorescence) and myeloperoxidase (MPO) specificity on ELISA. In contrast, in Asian populations, the majority of patients diagnosed with GPA have ANCAs that are mostly of the p-ANCA type and show MPO specificity on ELISA. Similarly, the incidence of GPA and MPA varies by geographical area and ethnicity (for example, MPA is more common in Asian populations) [49, 58, 59].

Clinically, the most distinctive features of GPA include granulomatous inflammation of the upper and lower respiratory tract [60], whereas EGPA is characterized by late-onset asthma, frequent non-infectious sinusitis, blood eosinophilia and tissue infiltrates with eosinophils and eosinophilic granulomas. The rhinitis and sinusitis in EGPA is often described as ‘allergic’, by contrast to infectious, and studies showed that around one-third of patients had documented allergies [61–63]. Patients with ANCA-positive EGPA more often present with vasculitic features such as renal involvement, pulmonary hemorrhage or mononeuritis multiplex but less frequently cardiac manifestations than their ANCA-negative counterparts [18]. Patients with MPA and GPA are at high risk of the development of both pauci-immune (or rapidly progressive) glomerulonephritis (40 to 80 %), alveolar hemorrhage (11 to 20 %) or both (pulmonary-renal syndrome). In contrast, glomerulonephritis and pulmonary hemorrhage may be rarer in patients with EGPA [64], with frequency varying with ANCA status. While in a recent study of 50 patients with EGPA the frequency of renal involvement and pulmonary hemorrhage seemed comparable to GPA and MPA (73 and 20 %, respectively), a previous and larger study demonstrated lower rates (27 and 7 %, respectively) [18].

The concept that patients with GPA or MPA should be rather and more simply classified by their ANCA status and type has been considered since the discovery of the ANCAs and continues to be supported by recent fundamental, genetic and clinical evidence [54, 65, 66] (Table 1). The pathogenic role of MPO-ANCAs has been demonstrated *in vitro* and in animal models and supported by a mother-to-fetus case report (although this remains an isolated case report), whereas that of PR3-ANCA is much less established [8, 67]. The predictive value of serial monitoring of ANCA titers remains controversial [68–71]. A meta-analysis demonstrated the ANCA titers do not reliably predict flares [68]. A recent study, however, showed that the predictive value of PR3-ANCA for disease flare could be better in those patients with GPA with renal involvement [72]. Recent genetic studies demonstrated that the association with the related single nucleotide polymorphisms *HLA-DP*, alpha-1-anti-trypsin (*SERPINA1*), and PR3 (*PRTN3*) were stronger for PR3-ANCA-associated disease than with a diagnosis of GPA established by physicians. Similarly, the association with *HLA-DQ* alleles was stronger with MPO-ANCAs than the clinical syndrome of MPA [54]. Finally, the outcomes of PR3- and MPO-ANCA-positive patients differ. Long-term mortality has been shown to be higher for MPO-ANCA- than PR3-ANCA-positive patients [73], although not in all studies [74]. In contrast, relapse rate is higher for PR3-ANCA-positive patients [66, 75, 76]. Histological classification of pauci-immune glomerulonephritis predicts renal outcome in GPA and MPA but does not add to other established poor prognostic factors, including increasing age, MPO-ANCA specificity, a high degree of tubular atrophy, and a low percentage of normal glomeruli [77, 78].

**Table 1:** Why the distinction between PR3- versus MPO-ANCA-positive (and versus ANCA-negative) vasculitides may be better than that based on the clinical diagnosis (GPA, MPA or EGPA)

● The reported frequencies of ANCA in GPA, MPA and EGPA and their specificities differ: for example, in non-Asian populations, GPA is mainly associated with PR3-ANCA (c-ANCA), and MPA and EGPA with MPO-ANCA (p-ANCA)
● The pathogenic role of MPO-ANCA is supported by *in vitro* experiments, animal models, and one (isolated) mother-to-inborn case report. The possible pathogenic role of PR3-ANCA is much less established yet
● The monitoring of PR3-ANCA is not a reliable marker of disease activity or predictor of flare, unless, perhaps, in those patients with renal involvement
● Genetic studies demonstrated that the associations with the SNPs of *HLA-DP*, *SERPINA1* and *PRTN3* with PR3-ANCA status and *HLA-DQ* alleles with MPO-ANCA status were stronger than with the diagnosis of GPA or MPA, respectively
● The outcomes of PR3- and MPO-ANCA-positive patients differ and the statistical associations between the ANCA type and renal outcome, mortality or relapse rates are stronger than with the diagnosis (GPA versus MPA)
● The exact place of EGPA in the ANCA-associated vasculitis group is unclear: the most common manifestations of EGPA (asthma, eosinophilia) are very different from those of GPA and MPA; less than 40 % of EGPA patients are ANCA-positive, mainly with MPO-ANCA (p-ANCA). None of the reported animal models of MPO-ANCA-associated vasculitis showed eosinophilic infiltrates as observed in EGPA

See text for detail and references. *ANCA* antineutrophil cytoplasm antibodies (c-ANCA refers to ANCA with a cytoplasmic labeling pattern in immunofluorescence, p-ANCA to ANCA with a perinuclear labeling pattern in immunofluorescence), *EGPA* eosinophilic granulomatosis with polyangiitis, *GPA* granulomatosis with polyangiitis, *HLA* human leukocyte antigen/histocompatibility antigens (DP and DQ are HLA class II antigen), *MPA* microscopic polyangiitis, *MPO* myeloperoxidase, *PR3* proteinase 3, *PRTN3* proteinase 3 gene, *SERPINA1* ‘serpin peptidase inhibitor, clade A, member 1’ coding for alpha-1 antitrypsin gene, *SNP* single nucleotide polymorphism

Currently, treatment of ANCA-associated vasculitis is not based on ANCA type. MPA or EGPA with a five-factor score of 0 (that is, proteinuria level <1 g/day, serum creatinine level <140 μmol/L, no cardiomyopathy, no severe gastrointestinal involvement and no CNS involvement) may be treated with glucocorticoids alone, with the addition of another immunosuppressive agent reserved for refractory disease or glucocorticoid-dependent patients. All GPA patients, as well as those with MPA or EGPA with five-factor score ≥1 and/or active renal disease, even if only renal-limited, should be treated with a combination of glucocorticoids and an immunosuppressive agent. For patients with localized, non-severe, non-renal GPA, methotrexate can be used. For more severe disease (that is, with renal, life- and/or organ-threatening manifestations), high-dose glucocorticoids must be combined with a more potent drug, such as cyclophosphamide, or rituximab for GPA or MPA [79–81]. Induction of remission in patients with relapsing GPA or MPA seems better with rituximab than cyclophosphamide, but in no other subgroups was either drug superior to the other [82]. Cyclophosphamide or rituximab at 18 months was also associated with similar rates of infection and other adverse events [82]. Compared with cyclophosphamide, however, the long-term use of rituximab is not associated with late bladder cancer or infertility. The addition of plasma exchange can be considered for progressive glomerulonephritis, alveolar hemorrhage or both, although the long-term benefits remain to be demonstrated [83, 84]. Once remission has been achieved, the options for maintenance treatment include azathioprine or methotrexate [85]. Leflunomide and mycophenolate mofetil are alternatives for patients who cannot tolerate the former two drugs (evidence for leflunomide efficacy is limited; mycophenolate mofetil is less effective than azathioprine for maintaining remission in GPA and MPA) [86]. The co-prescription of cotrimoxazole (at a higher dose than for *Pneumocystis jiroveci* prophylaxis) could further lower the risk of relapses [87]. Rituximab is likely another alternative for maintaining remission in GPA patients, but the optimal regimen remains to be determined, and it is not yet approved for this indication [88–90]. A recent randomized controlled trial demonstrated the superiority of rituximab infusions to azathioprine in maintenance therapy of GPA or MPA [91]. Other agents that may have a place in the therapeutic armamentarium for GPA include belimumab for maintaining remission (NCT01663623), abatacept for treating relapsing limited disease (NCT02108860) or gusperimus (NCT00530075). Mepolizumab may be useful for EGPA patients who experience multiple relapses and cannot stop glucocorticoids [92, 93].

Immune-complex small-sized-vessel vasculitides include a number of different diseases. Anti-GBM disease manifests clinically as pulmonary hemorrhage and acute kidney injury due to rapidly progressive necrotizing glomerulonephritis with linear deposits of Ig along the GBM. Renal prognosis is poor, with a frequent progression to end-stage renal disease, especially in patients with both ANCAs and anti-GBM antibodies, which occurs with variable frequency in 20 to 80 % in those with crescentic glomerulonepritis, usually with a preponderance of MPO-ANCAs [94–96]. The treatment relies on the combination of glucocorticoids, other immunosuppressants such as cyclophosphamide, and plasma exchange [95, 97]. Cryoglobulinemic vasculitis, not associated with hepatitis C virus (HCV) or lymphoma, can present skin, renal or peripheral nerve involvement. The treatment relies on the combination of glucocorticoids, immunosuppressive agents such as cyclophosphamide and/or, sometimes, plasma exchange or rituximab [98, 99]. IgA vasculitis is the most common vasculitis among children affecting boys more than girls, but can be seen in adults. Environmental agents and infections are commonly suspected as underlying triggers in children, but in adults indolent malignancy must be ruled out. The disease can manifest as rash, arthritis, and in severe cases nephritis, bowel edema or ischemia, and/or rarely with CNS involvement or alveolar hemorrhage. Several clinical trials have investigated the role of prednisone for IgA vasculitis but have failed to show benefit in preventing renal involvement [100–102]. While the role of glucocorticoids and other immunosuppressive agents for IgA vasculitis is unclear, they must be considered for severe or relapsing disease, in combination sometimes with an immunosuppressant [103, 104].

### Other vasculitides

Behçet’s disease can affect all types of vessels, arteries as well as veins. Epidemiologically, it is more common in Asian and Middle-Eastern populations [105]. Caucasian patients with Behçet’s disease are more often women, and they more frequently have gastrointestinal and neurologic involvement [106]. The treatment is adjusted on the basis of the type of manifestations and their severity and ranges from ‘simple’ drugs such as colchicine to much more potent immunosuppressive agents such as cyclophosphamide, anti-TNFα agents or alemtuzumab for severe forms. Apremilast is another drug that showed promising results in a recent phase II study in patients with Behçet’s disease and recurrent oral ulcers [107].

Cogan’s syndrome is a rare multisystem inflammatory disease, characterized by rapid onset of non-vasculitic ocular inflammatory lesions (pain, erythema, photophobia, blurred vision, interstitial keratitis) and inner ear symptoms (deafness, aural fullness, vertigo, tinnitus) [108]. Systemic symptoms occur in 50 % of patients and approximately 10 % develop associated large-vessel vasculitis manifested as coronary arteritis, aortitis, aortic aneurysms, aortic and/or mitral valvulitis, mesenteric vasculitis/thrombosis, and/or, rarely, glomerulonephritis. Its treatment is difficult and must be adapted to the disease severity [109].

Isolated CNS vasculitis is an extremely rare disease. Nonetheless, several subsets have been described in adults and children, including small- versus large-vessel CNS vasculitis, with different outcomes or responses to treatment [110, 111].

The treatment of vasculitides associated with probable etiologies or other systemic conditions must take into account the underlying condition. The treatment of HBV-associated vasculitis or HCV-associated cryoglobulinemic vasculitis must include antiviral drugs. Rheumatoid vasculitis should ideally rely on drugs effective for rheumatoid arthritis, including rituximab.

## Conclusion

### Will a future classification of vasculitides better match their therapeutic management?

Until revised and proficient tools become available, such as, perhaps, from the ACR-EULAR-Diagnosis and Classification of Vasculitis Study (DCVAS: NCT01066208), the 2012 CHCC nomenclature remains the most practical and main pragmatic way to differentiate and categorize vasculitides [2]. It parallels the current and different treatment strategies for those conditions: treatments for large-sized vessels remain closer to each other than for ANCA-associated vasculitides. However, compelling available evidence suggests that further changes may occur, including possibly a switch for ANCA-associated vasculitides from a clinical and phenotype-based classification to one based more on pathogenic mechanisms. As recently stated by Kemnaand Tervaert [5], ‘the classification of disease should focus not so much on the initial presentation, but on the biologic behavior of the disease’, including newly acquired knowledge of the prognosis, pathogenesis and genetics of vasculitides. Kallenberg [6] similarly concluded that ‘the ultimate intention is to base classification on aetiology and pathogenesis’.

DCVAS is supported by the ACR and EULAR and aims at developing a revised classification system and a validated set of diagnostic criteria for vasculitides [112]. Mainly based on the study of clinical manifestations at diagnosis of a large number of patients and controls, this effort will unlikely achieve a pathogenesis-based classification but may support different disease groupings and/or reveal subsets that may better parallel their pathogenesis.

A central question remains whether any changes in the currently used classification are really justified at this time. The identification of the causes of some of the vasculitides considered idiopathic or primary will obviously affect the future vasculitis classification and treatment, as it did for HBV- or HCV-associated vasculitides. The gradual identification of genetic vasculopathies that may have been ‘misdiagnosed’ as vasculitis (with mutations in adenosine deaminase 2 or in the gene encoding stimulator of interferon genes being the two most recently described entities) may reveal new pathways in the pathogenesis of inflammatory vascular disorders that can also justify changes in their grouping and treatments [51, 52, 113]. That said, prospective observational studies should be conducted before recommending future changes in the classification or nomenclature of vasculitides. Also, whether treatment strategies should differ by ANCA type or few genetic markers remains to be confirmed in well-designed controlled trials.

## Abbreviations

ACR: American College of Rheumatology
ANCA: antineutrophil cytoplasm antibody
CHCC: Chapel Hill Consensus Conference
CNS: Central nervous system
DCVAS: Diagnosis and Classification of Vasculitis Study
EGPA: Eosinophilic granulomatosis with polyangiitis
ELISA: Enzyme-linked immunosorbent assay
EMA: Medicines Evaluation Agency
EULAR: European League Against Rheumatism
GBM: Glomerular basement membrane
GCA: Giant cell arteritis
GPA: Granulomatosis with polyangiitis
HBV: Hepatitis B virus
HCV: Hepatitis C virus
IgA: Immunoglobulin A
IVIg: Intravenous Ig
LV-GCA: large-vessel giant cell arteritis
MPA: Microscopic polyangiitis
MPO: Myeloperoxidase
PAN: Polyarteritis nodosa
PR3: Proteinase 3
TAK: Takayasu arteritis
TNF: Tumor necrosis factor
